# Effects of uniform rocking motion on VLPO regional GABA/Glu contents and sleep in rats

**DOI:** 10.1007/s41105-025-00588-7

**Published:** 2025-06-23

**Authors:** Guang-yao Luo, Xian Jiang, Yu-lian Jin, Feng-ying Li, Ti-peng Wang, Bin Wen, Yao-wen Zhang

**Affiliations:** 1 Department of Otolaryngology, Head and Neck Surgery, Yanbian Hospital, Yanji, China; 2 Department of Otolaryngology, Head and Neck Surgery, Xinhua Hospital, Shanghai Jiaotong University School of Medicine, Shanghai, China

**Keywords:** Vestibular, Rocking, Sleep

## Abstract

Recent research has found that rocking promotes sleep in mice, but there is a lack of information on whether rocking also promotes sleep in rats and the neurophysiological mechanisms involved. The purpose of the present experiment was to elucidate the effects of rocking on sleep–wake staging in rats. EEG recordings were made on a platform with a frequency of 1.5 HZ and a displacement of 20 mm for 12 h of rocking and 12 h of stillness in rats, and the proportion of each sleep phase in the 24-h EEG was analyzed. The contents of glutamate and GABA in the VLPO region of rats were measured during shaking. The results showed that at a shaking frequency of 1.5 HZ, the proportion of non-rapid eye movement (REM) sleep increased and wakefulness decreased in the first 12 h of the rats, and there was no significant effect in the last 12 h. In the experiments, GABA levels in the VLPO region of awake rats gradually increased after the onset of uniform rocking exercise and began to decrease after reaching a peak at 20 min. After 40 min, GABA levels leveled off and did not change significantly above 60 min, but had no effect on glutamate levels. These results suggest that 1.5 HZ of rocking promotes NREM sleep and reduces wakefulness in rats, and that rocking may be related to GABAergic neurons in promoting sleep in rats.

## Introduction

The ventrolateral preoptic area (VLPO) is an important neural nucleus in the body that regulates sleep, body temperature, and energy dynamic balance, and plays an important role in sleep initiation, maintenance, and consolidation [1, 2]. There are many sleep-related neurons in the VLOP area, including GABA, GAL, and appetitive peptidergic neurons. VLPO projections to different sleep-promoting centers such as the tuberomammillary nucleus (TMN), ventral tegmental area, parafacial zone, and so on, and promotes sleep onset, the transformation of NREM sleep and REM sleep, and the important process of sleep to wakefulness [3]. Recent research has shown that astrocytes in the VLPO region can also promote sleep duration during the active phase in awake rats by optogenetic stimulation [4]. VLPO is one of the important nuclei of sleep.

The medial vestibular nucleus (MVN) is an important region of the vestibular autonomic reflex. Glutamatergic and GABAergic neurons are the main neuronal types in the medial vestibular nucleus of mice [5]. The medial vestibular nucleus receives afferents from the entire vestibular endings while sending a wide range of projections to the central nervous system. Recent research has demonstrated that fibers emanating from the MVN project to the circadian system as a sleep neuroreflex pathway [6]. In addition, areas projecting from the medial vestibular nucleus are thought to be involved in sleep and wakefulness. Horowitz and colleagues explore the destination of guinea pig vestibular afferent fibers using horseradish peroxidase and wheat germ agglutinin HRP anterograde axonal transport. Research has shown that the medial vestibular nucleus has a large number of nerve fibers projecting to areas that regulate wake–sleep-related functions, most of which project from neurons in the lateral part of the hypothalamus. This includes the locus coeruleus, covered cerebellopontine angle nucleus, nucleus raphes dorsalis, and the ventrolateral preoptic area [7]. The MVN and several sleep-related nuclei have rich neural pathways, which is one of the basic elements that can influence sleep, but it is not clear which nuclei play the main role.

The electroencephalogram (EEG) and electromyogram (EMG) are excellent biological indicators for studying sleep–wake states in rodents awakening, EEG shows low amplitude and fast frequency, while EMG shows active. During NREM sleep, the EEG is dominated by slower frequencies in the δ (0–4 Hz) and theta (4–7 Hz) ranges. EMG shows inhibition, during REM sleep. EEG shows abundant theta activity and EMG shows inhibition as in NREM sleep. The polysomnographic recording enables accurate determination of the sleep state of rats. In 1998, Quirarte measured the release of norepinephrine from the amygdala by foot electroshock using a microdialysis method [8]. Microdialysis is a technique for dynamic microbiological and biochemical sampling from living organisms, which, together with high-performance liquid chromatography analysis, enables accurate measurement of neurotransmitter levels in the extracellular fluid within the nucleus, and sampling *in vivo* can more closely resemble normal physiological conditions. For the fabrication of dialysis needles and the high-performance liquid phase mobile phase, Jin's experiments were chosen with reference [9].

As an important sensory system of the body, the vestibular organs play an important role in the sensory perception of spatial position and motor status [10]. There is a lack of research on vestibular autonomic reflexes. It has been shown that there are vestibular autonomic pathways in the central nervous system and that the vestibular nuclei have nerve fibers that project directly to autonomic nuclei in the brainstem to regulate heart rate, blood pressure, and respiration. And it also involves involvement in the control of complex behaviors such as sleep and wakefulness [11]. Early investigations of the vestibular–sleep relationship began in a sleep deprivation model, where long duration of sleep deprivation could lead to impairment of vestibular function and disturbance of balance in mice, while [12] sleep can reduce vertigo associated with chronic vertigo disorders [13]. It has now been shown that REM and NREM sleep are necessary for the process of neuroplasticity. This suggests that there is a correlation between sleep and vestibular compensation in patients with vestibular dysfunction. Rocking beds are widely used to promote sleep in infants. In recent research, it has been shown that the passive rhythmic movement of rocking is mediated through the vestibular otolithic organ and benefits sleep in humans and mice It was shown that rocking exercise shortened wake episodes and increased NREM sleep duration by accelerating sleep onset improved sleep architecture in mice and shifted EEG theta activity during wakefulness to slower frequencies [11]. They found that the optimal rocking rate for mice was 1.0 HZ and this sleep-promoting response was mediated by the otolith apparatus. In a recent controlled experiment, 60 subjects were subjected to rocking, and it was found that total sleep duration was prolonged in rocking subjects, N1 sleep duration was significantly reduced, N2 sleep duration was significantly increased, and N2 sleep stage latency was significantly reduced, suggesting that rocking promotes structural periods of human sleep [14]. The rocking motion during napping promotes the transition from wakefulness to sleep in humans [15]. Suwhan Baek and colleagues found that sleeping in a rocking recliner increases the duration of the spindle and deep sleep stages, thereby improving sleep quality [16]. Some studies have also found no significant change in the structure of rocking sleep [17], and it is worth noting that in the above studies, the environment as well as the parameters of the rocking platform were not the same, suggesting that there is a complex relationship between the intensity of rocking and the structure of sleep. All of the above demonstrate a clear correlation between sleep and vestibular function, but the specific neural pathway mechanisms have not been explored in depth. We found that rat rocking could promote NREM sleep in rats with uniform linear rocking motion at 1.5 HZ and did not affect sleep in the later 12 h. This pro-sleep response was mediated through the peripheral vestibular otolithic apparatus. Bilateral vestibular damage of rat rocking also had a tendency to promote sleep, and other effects such as proprioceptive, visual for sleep cannot be excluded. In the present study, we first observed whether shaking stimulation induced changes in the sleep phases in rats, and whether vestibule mediate this pro-sleep, and measured the levels of GABA and glu in the VLPO area during shaking in normal rats and vestibular function rats. The neurons involved, and the underlying neural pathways, were indirectly inferred by observing the neurons that cause excitation through microdialysis.

## Materials and methods

### Animals and surgical procedures

#### Animals

Male SD rats of about 200 g were selected and all rats were housed individually before and after surgery at a temperature of 22 ± 3°C and relative humidity of 56 ± 5% in a suitable ventilated environment with adequate water and food, unrestricted activity, and light and dark conditions, provided by the Experimental Animal Center of Yanbian University. Experimental procedures were carried out in accordance with the Animal Protection and Utilization Committee of Yanbian University.

#### Surgery

Rats were anesthetized with pentobarbital solution (50 mg/kg, i.p.), fixed in a brain stereotaxic instrument. The bregma was fully exposed and the electrodes were buried in EEG(+) ml:-1.5mm Ap-1.0:EEG(-):ml1.5mm AP-2.0 (ground) ml:-i.5 AP-3.0. silver wire electrodes were inserted into the neck muscles. The miniplug was connected, the wiring was checked for short circuits, and later the plug was covered and fixed with dental cement. After EEG implantation, rats were given 5 days to acclimatize to the electrode implantation and 1 day to acclimatize to the environment in which the cables are connected and tested to ensure stability of the test.

### Experimental procedure

#### EEG measurements

Microdialysis** the control rocking group (*n* = 7) recovered from surgical use of EEG electrode implantation for 5 days, took the lead in the rocking cage for 1 day to acclimatize, rocked at 8:00 am the next day, and then 24-h EEG was performed after 12 h of rocking. In the vestibular injury rocking group (*n* = 7), 100 μL of sodium arsanilate (100 mg/mL) was intratympanically injected into the left middle ear of the rat, and auricular twitching was considered as a successful injection, and the EEG electrode was implanted for 5 days after 7 days of recovery for surgical adaptation, and vestibular function was measured for 12 days. After 1 day of acclimatization in the rocking cage, rocking was performed for 12 h and EEG was measured continuously for 24 h. In the control stillness group (*n* = 6), the EEG electrodes were placed in the rocking cage for 24-h EEG measurements 5 days after adaptation to surgical use, and in the vestibular damage stillness group (*n* = 6), the same surgical protocol was used as in the vestibular injury rocking group, and 24-h EEG measurements were performed 1 day after adaptation to the rocking cage.

**Fig. 1:**
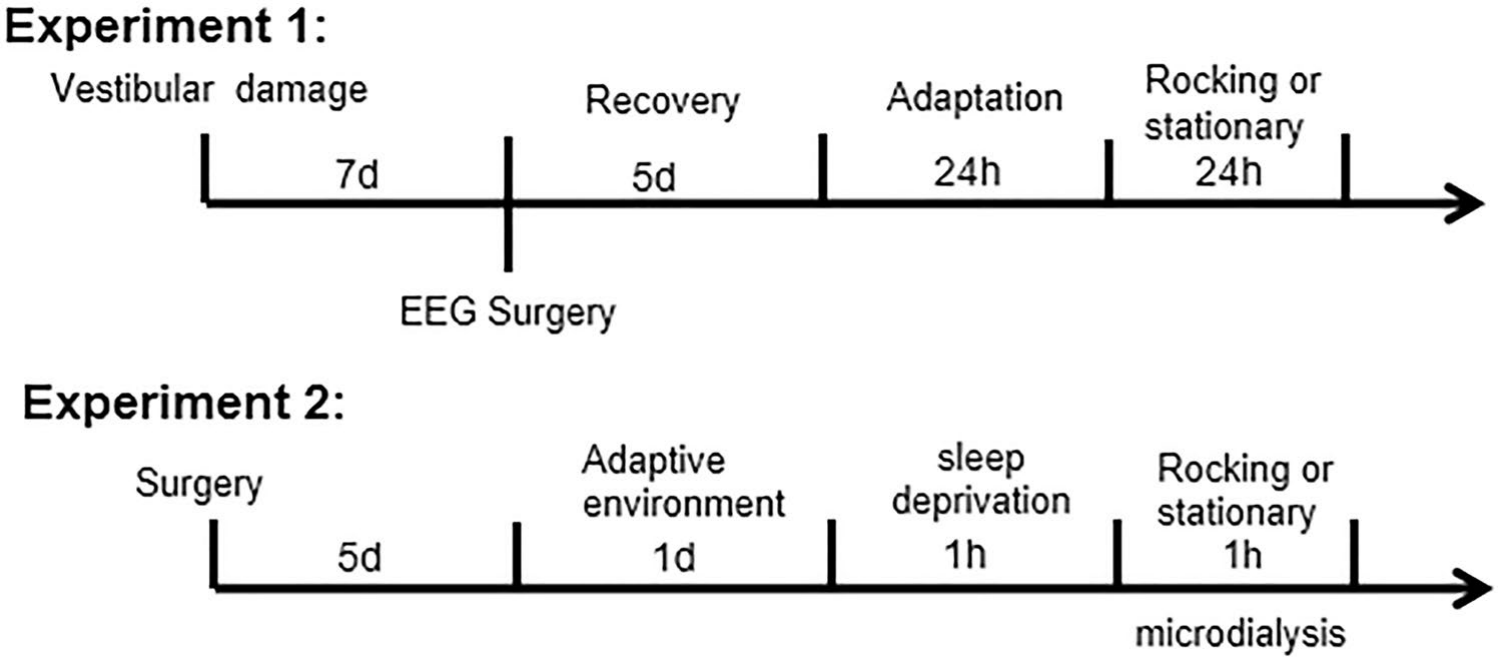
Experimental flow chart

To observe the changes of neurotransmitters in the vestibular nucleus of rats by uniform rocking motion, we divided the rats into 2 groups, namely control group (*n* = 6) and rocking group (*n* = 6), and implanted microdialysis needles into the VLPO of the ventral lateral nucleus of rats (AP: 0.12mm ML:0.1mm DV:9.0mm) to dialyze the neurotransmitters in the VLPO area by rocking. Rats were executed after dialysis to see if the dialysis needle was in the VLPO area, those not in the VLPO area were not counted. Rats recovered for 5 days after dialysis needle implantation, adapted to the dialysis environment for a day. On the second day, 1-h sleep deprivation was performed before shaking. Modified Ringer’s solution was perfumed at a constant rate of 1.5 μL/min during shaking, and dialysis fluid from the VLPO area was injected into a high-performance liquid-phase detection system (HTEC- 500 EICOM, Japan) to analyze the neurotransmitter content (Fig. 1)

#### Behavioral experiments

To observe whether the vestibule was disrupted, we used head bobbing, circling, retropulsion, tail-hanging reflex, contact-inhibition reflex, and air-righting reflex for scaling 0 to 4 scores [16]. Head bobbing include rotation, backward and abnormal head movements. Repeated circling motion, retropulsion displacement of the body backward, tail-hanging reflex are the instinctive responses to lifting the rat’s tail, with the rat’s arms extended in a ready-to-land position. The abnormal response is the rat’s abdominal curl or the rat “crawling” toward the tail as a dorsal “landing”. Contact-inhibition reflex is when a rat is placed on its back on a horizontal plane and a metal fence is placed in contact with the bottom of the rat’s feet, the normal rat will quickly roll over, but abnormal rats will “walk” in the opposite direction because they have no sense of direction, lying on their backs with their feet facing upward. The air-righting reflex involves the animals remaining supine and falling from a height of 40 cm onto a cushion. Normal rats were able to successfully straighten itself in the air, while vestibular-deficient rats were not. An average score was calculated for all behaviors to give a score for vestibular function.

### Experimental equipment

The experimental platform used HY- 5B gyratory oscillator (Changzhou Hongze Experimental Science and Technology Company, China), which can provide 20 mm displacement distance and can adjust the swinging frequency, and the EEG experiments were performed using the labchart data acquisition and analysis system (Australia), with a sampling frequency of 1k/s, which performs the discrete Fourier transform of brain waves and generates the power spectrum, with a measurement range of 0.2–50 Hz. The noise of the experimental environment was maintained below 60 dB.

### Statistical analysis

Data were processed using SPSS 17.0 statistical software, and GABA and Glu release in the VLPO area at the beginning of the uniform rocking motion was used as the base value to calculate the percentage of GABA and Glu content from the base value in each case sample. EEG data were spectrally analyzed using fast Fourier transform, and NREM sleep, REM sleep, and wakefulness states were semi-automatically classified for each 20-s period using a customized sleep analysis software (lunion stage). Brain state classification was manually validated after automatic scoring. Data are expressed as mean±SE, and statistics were performed by applying one-way ANOVA and group comparison t test, with the level of significant difference set at *P* < 0.05.

## Result

**Fig. 2:**
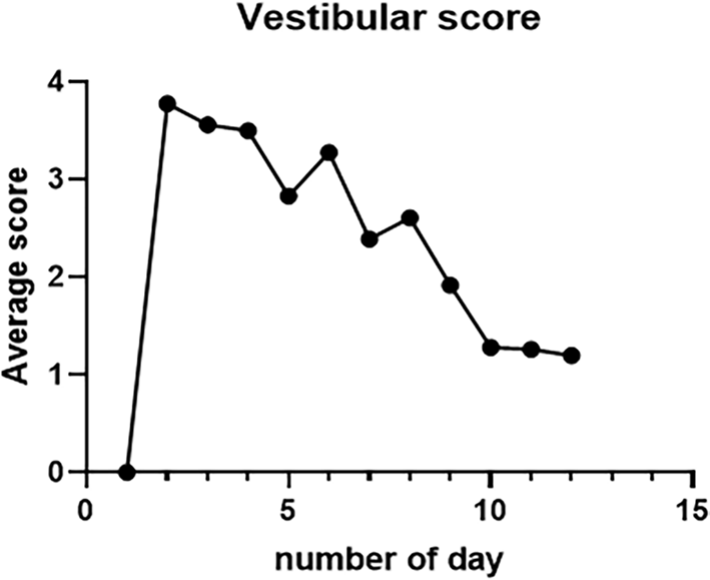
Vestibular damage rats, 12-day vestibular score

In rats injected with arsanilate in the middle ear bilaterally, the vestibular function scores changed over 12 days (*n* = 8), with scores ranging from 1 to 4. The higher the score, the more severe the vestibular impairment, with symptoms peaking on the second day with vestibular destruction, and gradually plateauing towards normal vestibular function on day 12 (Fig. 2).

**Fig. 3:**
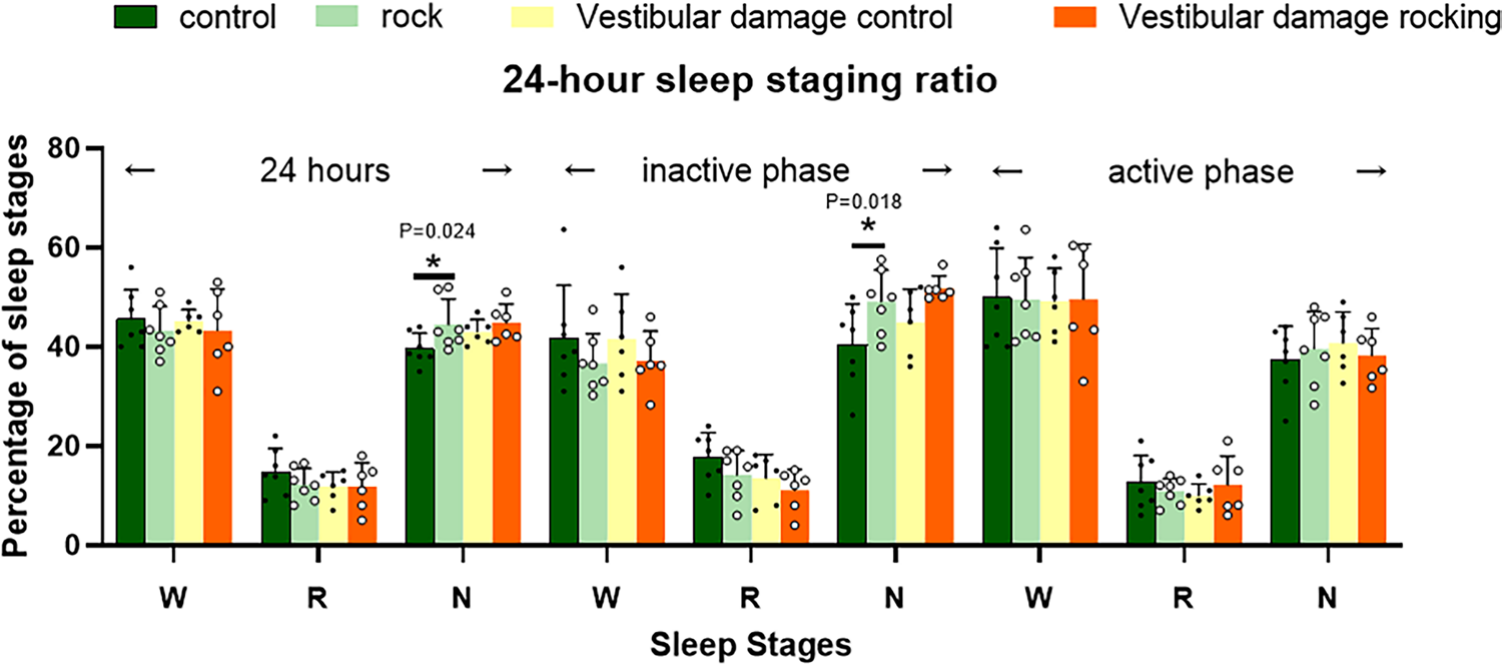
The proportion of each phase of 24-h EEG

Since rodents are nocturnal, we defined the rodent physiological sleep time period from 7 am to 7 pm as the inactive period, and the rodent physiological activity period from 7 p.m. to 7 a.m. as the active period. 24-h EEG showed NREM sleep with more percentage of sleep in the swing group than in the control group (*P* = 0.024 < 0.05). The first 12 h of swaying NREM sleep accounted for a greater percentage of sleep than the control group (*P* = 0.018<0.05). There was no difference between the 4 groups in the post 12 h, indicating that 12 h of stimulation at 1.5 HZ frequency with 20-mm displacement did not affect sleep in the post 12 h. There was no statistical significance between the vestibular damage control and vestibular damage sway groups, and we also demonstrated that sway stimulation for sleep requires the involvement of the vestibular organs. This is similar to the results of Kompotis K. [11] experiments (Fig. 3).

**Fig. 4:**
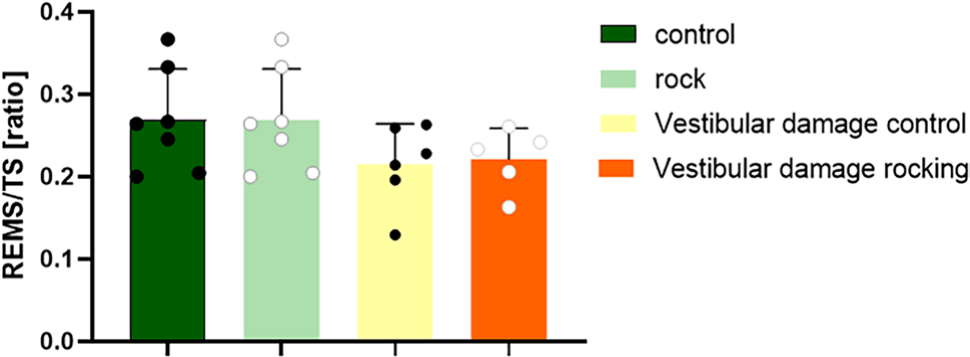
REM sleep as a percentage of total sleep

In mice, 1.0 and 1.5 Hz primarily promoted NREM sleep at the expense of wakefulness and REM sleep (Fig. 4).

Effect of uniform rocking exercise on GABA content in VLPO area of awake rats

**Fig. 5:**
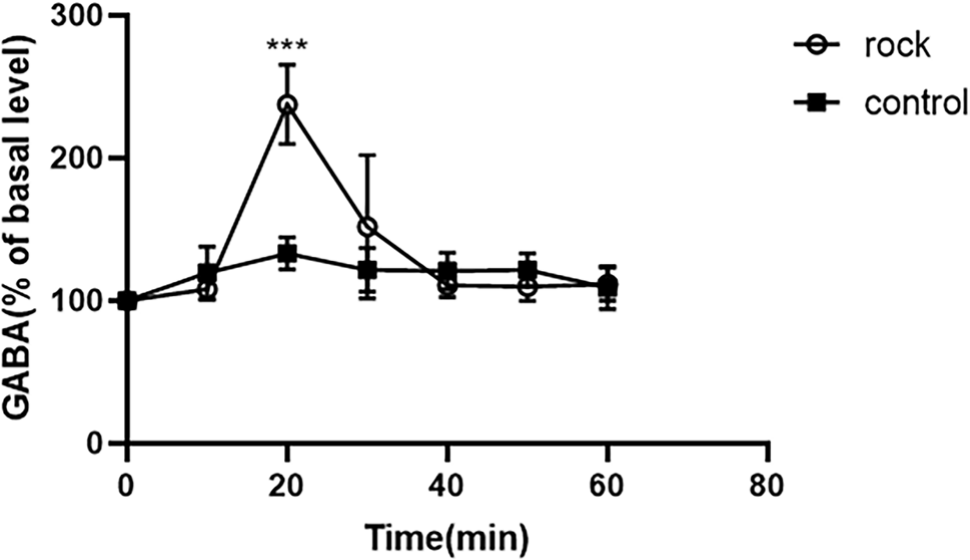
Effect of uniform rocking motion on GABA content in the VLPO area of stationary rats

The GABA content in the VLPO area of normal awake rats gradually increased after the start of uniform rocking exercise and began to decline after reaching a peak at 20 min, and the change of GABA content basically stabilized after 40 min, and no significant change was observed above 60 min (Fig. 5).

**Fig. 6:**
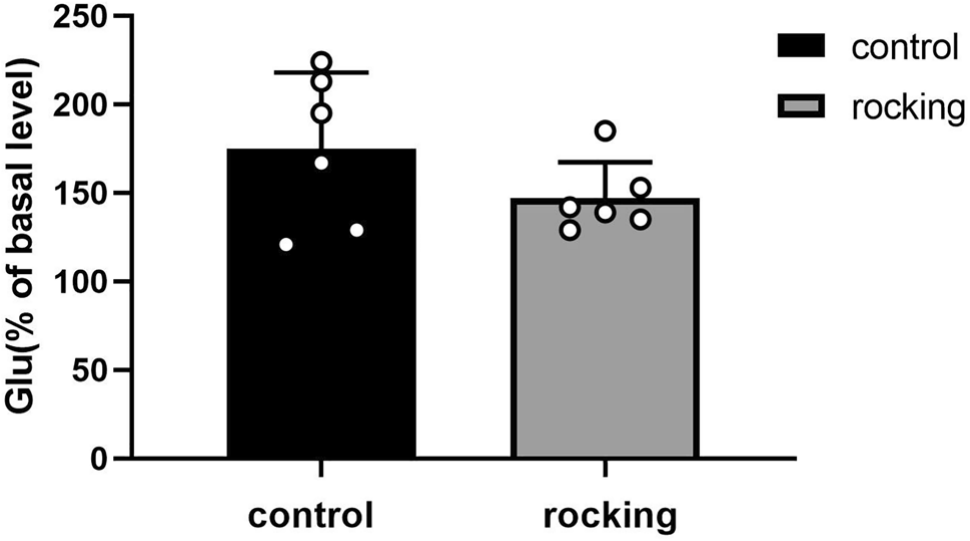
Effect of uniform rocking motion on Glu content in the VLPO region

There was no statistical difference in the Glu content in VLPO between the experimental group of stationary rats after the start of uniform rocking exercise, and the group of rocking rats (as shown in Fig. 6).

## Discussion

The polysomnography (PSG) system can visually determine the sleep and wakefulness status of rats by EEG. In this experiment, after the rats were given 1.5 HZ uniform rocking motion, the normal rocking group had more NREM sleep and less wakefulness than the vestibular disrupted rocking group, which indicated that the sleep-promoting effect caused by uniform rocking motion was felt by the peripheral vestibular receptors and transmitted by the vestibular nerve pathway, and there was a clear neural pathway between the vestibular and sleep-promoting centers. However, balance is the result of the interplay of peripheral vestibular, visual, and proprioceptive senses, and it cannot be ruled out that visual and proprioceptive senses can respond to swaying, and the subsequent role of visual and proprioceptive senses on the vestibule needs to be considered independently. This is clearly shown as the ratio of REM sleep to total sleep (TS) [11]. We have not replicated the observation of 1.5 Hz sway in mice, probably due to different mechanical characteristics of the vestibular organs. The otolithic membrane can generate shear forces under gravity or inertia that cause cilia to oscillate, following which the cilia of the vestibular hair cells are deflected by tugging, the cellular ion channels open or close, and then signals are transmitted synaptically to the vestibular nerves, which are detected by the brain to perceive movement. Although mice and rats are the same species, they differ in the area of the otolithic membrane, the number of hair cells, and the size of the hair cells [18]. Acceleration is the same, but the otolith organs do not receive the same inertial forces, and the brain receives neural stimuli differently, so the optimal rocking frequencies of mice and rats are likely to differ. Although the results for mice were not replicated in rats, the results for sleep promotion still represent that 178 cm/s^2^ is still in the optimal range for sleep promotion.

The microdialysis results showed that the content of GABA in the VLPO area began to increase 10 min after the start of rocking movement, peaked after 20 min and began to decrease, and reached the basal value at 40 min. However, there was a slight difference with the time of entering sleep in EEG rats. Rocking to promote sleep may be associated with a transient increase in VLPO, but no significant change in Glu occurred, suggesting that rocking may not be associated with glutamatergic neurons within VLPO.

The increase in GABA content in the VLPO area during uniform rocking movements may be related to the inhibition of its postganglionic neuronal activity, and the EEG wave asynchrony may be related to the possible existence of other neural mechanisms in the ventral lateral preoptic area. Recently, it has been demonstrated that firing of GABAergic neurons in the LMVN (medial vestibular nucleus) underlies stable wakefulness and smooth transition from non-REM sleep to fast sleep, and viral tracing was used to show a direct link between the GABAergic vestibular nucleus and VLPO. This explains rocking can affect GABAergic neurons [19]. Other potential pathways may also indirectly affect sleep. For example, the sleep-promoting mechanisms of AD are in the dorsal forebrain (BF), cerebral cortex and hippocampus, and extracellular levels of AD increase with awake and down time during sleep extending [20]. AD levels are regulated by AD kinase in astrocytes [18, 21]. Injection of AD receptor agonists into the brain increases NREM sleep, while AD antagonists (e.g., caffeine) promote awakening [22]. Rocking may cause sleep by increasing the accumulation of AD in the sleep nucleus. Bilateral vestibular damage in rats can significantly induce theta wave power [23], and patients with bilateral vestibular damage were significantly anxious, but significant reduction in anxiety levels after moderate galvanic vestibular stimulation treatment in healthy young adults [24]. This confirms the modulatory effect of vestibular stimulation on mood state, emotional control, and anxiety levels. This effect of anxiolytic levels may indirectly promote an increase in sleep with a decrease in sleep latency time.

Glu and GABA are among the important neurotransmitters in the central nervous system that regulate sleep–wake, and are important in the normal physiological state of the body [25]. Glu is a widely distributed excitatory neurotransmitter in the brain, which has an important role in maintaining the excitatory state of the cerebral cortex, and we expect its alteration in the constant rocking movement state to be different corresponding to alteration of inhibitory neurotransmitter GABA. The excitatory neurotransmitter Glu content in this experiment did not show significant changes in normal awake rats during peripheral vestibular stimulation and in the group without peripheral vestibular stimulation, so the specific mechanism of whether Glu delivers nerve impulses to vestibular receptors in the vestibular nerve conduction pathway and exerts excitatory effects in the neuromodulatory axis needs to be further investigated. It may be related to the low distribution of Glu neurons in VLPO, but it cannot be excluded that in other nuclei, Glu improves sleep by swaying, and sleep is produced by the joint action of multiple neurotransmitters in multiple nuclei, disrupting neurons in individual parts of the ascending wakefulness system, including cholinergic neurons in the basal forebrain, or multiple neurons such as (basal forebrain cholinergic neurons, orexins neurons and locus coeruleus neurons) also do not have a significant effect on the amount of waking sleep [26, 27]. Therefore, it needs to be further investigated in relation to several neural clusters, such as VLPO projecting to locus coeruleus, histaminergic neurons in the posterior hypothalamic nodal papillary nucleus (TMN), cholinergic neurons in the lateral dorsal tegmental nucleus of the pons, and 5-hydroxytryptaminergic neurons in the dorsal nucleus of the middle suture. These projected areas are inextricably linked to sleep [6]. VLPO active nerves are mainly GABAergic neurons, while GABA neurons of VLPO have subtypes that produce Galanin [28] which is also closely related to sleep [29]. The specific GABAergic neurons in VLPO are able to promote NREM sleep and reduce wakefulness [30], which is similar to our results.

Insomnia can cause decreased quality of life, fatigue, bad moods, decreased learning and memory functions, decreased immune function, and even lead to various diseases. For people with insomnia, there is no proven method other than taking benzodiazepine anti-insomnia medications. Although there have been many studies on sleep stimulation, there are few data to support the use of non-pharmacological treatments for insomnia. Our study shows that moderate amounts of homogeneous rocking movement stimulation can cause an increase in the content of the inhibitory neurotransmitter GABA in the VLPO region, effectively promoting sleep, and the results can be expected to bring value to the combined clinical application.
